# Sex, drugs, risk and resilience: analysis of data from the Canadian Health Behaviour in School-aged Children (HBSC) study

**DOI:** 10.1093/eurpub/cky169

**Published:** 2018-09-04

**Authors:** Susan P Phillips, Nathan King, Valerie Michaelson, William Pickett

**Affiliations:** 1 Department of Public Health Sciences, Queen’s University, Kingston, Ontario, Canada; 2 Department of Family Medicine, Queen’s University, Kingston, Ontario, Canada; 3 Department of Religious Studies, Queen's University, Kingston, Ontario, Canada

## Abstract

**Background:**

Risk-taking behaviour among adolescents, particularly those experiencing childhood adversities, can predispose to injury, unwanted pregnancy, long-term morbidity and death. Resilience, i.e. adapting to threats and thriving, has rarely been examined as a protective factor for adolescent risk-taking. We studied whether the malleable traits of empathy, confidence, self-control and optimism, all markers of resilience, align with decreased risk-taking despite adversity, among 11–15 year olds.

**Methods:**

From responses of 22 643 Canadian youth to the Health Behaviour in School-aged Children (2014) survey we validated a five-item resilience scale. Using regression analyses, this scale and a single measure of self-control were considered as potential protective factors for a composite measure of risk-taking behaviour and of initiation of sexual activity before age 14.

**Results:**

There was a dose-dependent association between greater resilience and diminished risk-taking for boys and, even more so, among girls. This relationship remained significant after controlling for family and social support, implying that greater resilience may override the detrimental impact of childhood adversity on risk-taking. The least resilient youth were most likely to report early sexual activity, although this relationship was not linear. Generally, the impact of self-control on risk-taking was not statistically significant, perhaps because of shortcomings of the self-control indicator.

**Conclusion:**

Brief screening protocols can identify assets that protect against risk-taking behaviours among adolescents. The malleable nature of these traits offers primary care providers and public health personnel a novel and effective route to decreasing adolescent risk-taking and fostering future health.

## Introduction

Adolescent substance use, violence or fighting and early sexual activity pose immediate health risks including injury, overdose, unintended pregnancy and sexually transmitted infection, and may foreshadow long-term mental and physical illness.^1^ Adversities such as poverty, disrupted families or exposure to violence also are correlated with adolescent risk-taking behaviour.^2^^,^^3^ Unfortunately, methods for modifying or ameliorating these adversities at the individual or population level are elusive and politically controversial.^1^ Ongoing clinical, public health and policy efforts to educate and prevent risk-taking have not dislodged injury as the primary cause of death among adolescents.^4^^,^^5^ We wished to step back from the outcome of risk-taking behaviour and examine whether there are modifiable personal characteristics that buffer adversity and safeguard health by diminishing adolescent risk-taking. In particular, we propose resilience as such an asset, one that focuses on strengths rather than deficits and that can be measured using a variety of scales.

Clinical medicine is only beginning to consider assessing and fostering individual strengths and building on decades of research demonstrating that positive health effects can stem from particular personal attributes.^6–8^ Might the characteristics that collectively build resilience also hinder risk-taking behaviour and counter some of the harms arising from childhood adversity?^9^ Resilience is defined differently by different authors.^10–12^ Most include constructs of exposure to adversity and positive adjustment that result from a set of individual assets.^13–16^ These include empathy, self-control, optimism/sense of meaning and self-confidence/self-efficacy. Such characteristics are malleable, can be fostered by individual efforts, supportive family and social environments,^17^ and appear to protect against risk-taking among 13–16 year olds,^18^ older adolescents^16^ and university students.^19^ Existing evidence suggests this relationship differs for boys and girls and is neither consistent nor incremental.^1^^,^^18^ In addition, there is ambiguity as to whether greater resilience is associated with healthier adolescent behaviour despite childhood adversity. We wished to explore this to determine whether modifiable personal assets and characteristics predict the risk-taking that predisposes to injury and diminished long-term health among youth. Using Canadian data from the Health Behaviour in School-aged Children (HBSC) study we examined whether, despite childhood adversities, the personal assets of empathy, self-confidence, self-control and optimism, commonly considered as markers of resilience, had a protective effect on risk-taking among youth aged 11–15.

## Methods

### HBSC dataset

Data were from the 2014 (Cycle 7) HBSC study (the Canadian portion of the WHO’s 47 nation survey of youth health) of 29 837 students in grades 6–10 at 369 schools across Canada.^20^ Sampling was stratified within Canadian regions by language, school type (public or Catholic) and community size. Excluded were private and special schools and those on Indigenous reserves (<7% of eligible student population).^21^ Sampling weights ensured proportional provincial and territorial representation. Self-reporting questionnaires were administered in school classes.

After restricting the sample to students with complete data for the relevant variables, sample size was 22 643 (weighted *n* = 22 964; 10 889 boys and 12 075 girls). Sexual behaviour questions were asked of grade 9–10 students only, limiting analyses of this outcome to a subgroup (weighted *n* = 7838; 3748 boys and 4090 girls).

### Ethics

Ethics approval for the HBSC study was granted by the General Research Ethics Board at Queen’s University (GMISC-062-13), the Public Health Agency of Canada and Health Canada's Research Ethics Board. Participation was voluntary, and consent (explicit or implicit depending on local protocol) was obtained from school administrators, parents and participating students.

### Indicators

#### Resilience

A literature review identified three commonly used adolescent resilience scales and one review article of particular utility.^13–16^ We compared components of individual resilience across these, mapping common concepts onto each other when terminology differed. Empathy, self-control, confidence/competence and sense of meaning/optimism appeared in two of three scales.^22^ We then identified the following seven HBSC questions as potential indicators of these components for our derived composite measure of resilience:

I often help people without being asked (empathy)

I try to be kind to other people (empathy)

I have confidence in myself (personal competence/confidence)

I feel that my life has meaning or purpose (sense of meaning/optimism)

Cantril ladder of best to worst possible life (optimism)

*I have trouble making decisions (confidence/self-control)

*I have a hard time saying no (self-control)

*items removed from final composite resilience measure

The original questions in the HBSC were measured with different scales. Based on precedent, we re-categorized a number of ordinal and continuous outcomes into three levels, consisting of 0 (minimal) to 2 (greatest), by combining positive, neutral and then negative responses. These could then be reasonably explored as a summative, composite scale of resilience using exploratory factor analyses for such categorical data.^23^ We were developing a new scale from a novel data source and, *a priori*, we did not have strong hypotheses about the number of factors that might contribute to this scale, nor how the items would load onto one or more ‘factors’. This made exploratory factor analysis appropriate as a means of exploring the underlying factor structure and reducing the number of items to only those that loaded onto the factor of interest (‘resilience’). Based on maximum likelihood estimation and the eigenvalue cut-off of 1, a single factor solution fit the data. The lowest factor loading item was then sequentially removed until all remaining loaded onto the factor above an acceptable cut-point (factor loadings for retained items= 0.34–0.79).^24^ As indicated above, this left five items in the composite scale (Cronbach’s alpha= 0.63) giving each participant a potential score of 0 (least) to10 (most resilient). The scale was categorized into five groups: group 1 i.e. least resilient (score 0–4), group 2 (score 5–6), group 3 (score 7–8), group 4 (score of 9), group 5 (score of 10). Because of its importance as an indicator of resilience we also explored the single, specific self-control question as a separate measure.

#### Outcomes

Risk-taking was measured as: (i) a composite of overt risk-taking and (ii) an individual item describing early (and assumed risky) sexual behaviour.

*Overt risk-taking* was measured using a factor analytically derived scale (Cronbach’s alpha= 0.75) combining smoking history, use of alternative tobacco products, frequency of alcohol consumption, drunkenness, no bike helmet use, physical fighting and caffeinated energy drink consumption.^25^ Behaviours were labelled as minimal (0), moderate (1) or frequent (2), then combined using standardized weights from exploratory factor analysis. Overt risk-taking was dichotomized [top quartile (greatest risk-takers) vs. the rest]. This scale was developed and validated among grade 6–8 students, but was applied to the full sample (grade 6–10 students).

The sexual behaviour indicator used the question: ‘How old were you when you had sexual intercourse for the first time?’ Responses of 13 or younger were considered as evidence of risk-taking.

#### Confounders

We considered numerous potential confounders including age, biological sex and socioeconomic status (SES) assessed via the validated question, ‘How well off do you think your family is?’ (‘Well-off’, ‘Average’ and ‘Not well off’).^26^ We also included measures of contextual environment using previously validated scales. Family social climate was assessed with a four item scale (*α* = 0.90).^27^ A five-item scale indicated community support and social capital (*α* = 0.79).^28^ Finally, perception of school climate used a four item scale (*α* = 0.79).^20^ Each covariate was categorized into ‘high’, ‘medium’ and ‘low’, using tertiles based on the sample distribution. Individual items and the psychometric origins/properties of the family social climate, social capital and school climate scales are available at: http://healthycanadians.gc.ca/publications/science-research-sciences-recherches/health-behaviour-children-canada-2015-comportements-sante-jeunes/index-eng.php.

### Statistical analysis

All analyses used SAS Version 9.4, were sex stratified, and included sampling weights. The continuous measure of resilience used measures of central tendency and variability. One-way ANOVA was used to test whether resilience scores differed between boys and girls or by the covariates considered, adjusting for clustering by school via Generalized Estimating Equations.

A series of log-binomial regression models examined the association between the resilience scale or self-control (primary exposures), and risk-taking (primary outcome). Model 1 estimated unadjusted relative risks. Model 2 was adjusted for age and SES, while Model 3 was adjusted further for family and community support. A similar approach was used to examine the association between resilience or self-control, and engagement in early sexual behaviour. Because this sample had a narrow age range (grade 9–10), we did not adjust for age, and community support was not included in the final adjusted model for girls because of non-convergence issues.

## Results

### Resilience

Overall, participants appeared to be relatively resilient with mean scores ranging from 6.6 to 8.8 (maximum= 10). These scores varied with individual and social circumstances. Greater resilience was seen among boys, younger participants, and those of highest SES (table 1). Family support, community support and more optimal school climate, all markers of positive and supportive environments, were also strongly aligned with higher resilience. These relationships were similar for boys and girls, although resilience scores varied more widely among girls.

**Table 1:** Description of resilience score (range= 0 or least resilient to 10 or most resilient)

	Boys			Girls		
	*n*	Mean	(SD)	*n*	Mean	(SD)
Overall	10 889	8.0	(1.9)	12 075	7.8	(2.0)*
Age						
≤11	992	8.2	(1.8)	1074	8.2	(1.9)
12	1907	8.1	(1.8)	2060	8.1	(2.0)
13	2129	8.1	(1.9)	2309	7.8	(2.1)
14	2353	7.9	(1.8)	2792	7.6	(2.1)
≥15	3508	7.8	(2.0)	3840	7.6	(1.9)
		*P-trend <0.0001*			*P-trend <0.0001*	
Socioeconomic status						
Well-off	6415	8.3	(1.7)	6493	8.3	(1.8)
Average	3601	7.6	(1.9)	4371	7.4	(1.9)
Not well-off	873	7.0	(2.2)	1210	6.5	(2.3)
		*P-trend <0.0001*			*P-trend <0.0001*	
Family support						
High	4283	8.8	(1.4)	4385	8.8	(1.4)
Medium	4002	8.0	(1.6)	4001	8.0	(1.6)
Low	2604	6.6	(2.1)	3689	6.3	(2.1)
		*P-trend <0.0001*			*P-trend <0.0001*	
Community support						
High	4002	8.6	(1.7)	3791	8.6	(1.5)
Medium	3781	8.0	(1.7)	4377	7.8	(1.9)
Low	3107	7.1	(2.2)	3906	6.9	(2.2)
		*P-trend <0.0001*			*P-trend <0.0001*	
School climate						
High	3498	8.8	(1.4)	3809	8.8	(1.4)
Medium	3815	8.1	(1.6)	4116	8.0	(1.7)
Low	3297	7.0	(2.2)	3919	6.6	(2.2)
*Missing*	*280*			*231*		
		*P-trend <0.0001*			*P-trend <0.0001*	

All values are weighted; SD=standard deviation.

* Significant difference in overall mean resilience score comparing boys and girls (*P*<0.0001).

### Resilience and risk-taking

Increments in resilience, particularly when measured using the composite scale but also for the single measure of self-control, were consistently associated with reductions in reported risk-taking across age groups (see table 2), for boys and girls, and with evidence of statistical significance, and of a dose-response relationship. Among the most resilient boys, 19% were in the highest quartile of risk-taking while for the least resilient this proportion was 50.4%, a 31.4% absolute difference. The pattern was even more striking in girls with a change from 10.6% in the most to 52.5% in the least resilient group (41.9% absolute difference). After adjusting for age, SES, family support and community support, the least resilient boys were still 1.98 times more likely (95% CI: 1.59–2.46) and least resilient girls were 2.68 times more likely (95% CI: 2.18–3.31) than the most resilient to engage in risk-taking. The impact of low self-control on greater risk-taking showed a similar incremental pattern for boys (RR= 1.17; 95% CI: 1.05–1.31) and girls (RR= 1.10; 95% CI: 0.98–1.23) although among girls this finding lacked statistical significance.

**Table 2:** Log-binomial regression estimating relative risk of engaging in overt risk-taking behaviour (top quartile) by sex, grades 6–10

	*N* total	High risk-taking		Model 1^a^		Model 2^b^		Model 3^c^	
		*n*	(%)	RR	(95% CI)	RR	(95% CI)	RR	(95% CI)
Boys (*n*=10 889)									
Resilience									
5 (highest)	2420	460	(19.0)	1.00	–	1.00	–	1.00	–
4	2904	614	(21.1)	1.12	(0.94–1.19)	1.07	(0.91–1.26)	1.03	(0.87–1.22)
3	3419	1022	(29.9)	1.57	(1.28–1.91)	1.44	(1.18–1.76)	1.34	(1.09–1.64)
2	1461	566	(38.7)	2.00	(1.63–2.46)	1.73	(1.41–2.12)	1.57	(1.27–1.93)
1 (lowest)	684	345	(50.4)	2.62	(2.11–3.25)	2.25	(1.84–2.76)	1.98	(1.59–2.46)
Self-control									
High	4744	1164	(24.5)	1.00	–	1.00	–	1.00	–
Moderate	2927	828	(28.3)	1.07	(0.97–1.19)	1.07	(0.97–1.17)	1.05	(0.95–1.16)
Low	3218	1014	(31.5)	1.20	(1.07–1.36)	1.18	(1.06–1.32)	1.17	(1.05–1.31)
Girls (*n*=12 075)									
Resilience									
5 (highest)	2566	273	(10.6)	1.00	–	1.00	–	1.00	–
4	2727	449	(16.5)	1.54	(1.23–1.92)	1.32	(1.06–1.63)	1.23	(1.00–1.51)
3	3995	985	(24.7)	2.28	(1.79–2.90)	1.78	(1.42–2.24)	1.57	(1.28–1.92)
2	1853	738	(39.8)	3.63	(2.82–4.68)	2.62	(2.06–3.32)	2.14	(1.75–2.62)
1 (lowest)	933	490	(52.5)	4.75	(3.70–6.10)	3.38	(2.67–4.29)	2.68	(2.18–3.31)
Self-control									
High	4645	940	(20.2)	1.00	–	1.00	–	1.00	–
Moderate	3209	761	(23.7)	1.05	(0.92–1.20)	1.08	(0.96–1.21)	1.07	(0.95–1.20)
Low	4220	1233	(29.2)	1.18	(1.04–1.35)	1.11	(0.99–1.24)	1.10	(0.98–1.23)

RR = relative risk; 95% CI = 95% confidence interval; adjusted for clustering by school and weighted.

a Model 1=unadjusted (resilience and self-control in the same model).

b Model 2=adjusted for age, and socioeconomic status.

c Model 3 =adjusted for age, socioeconomic status, family support and community support.

### Resilience and early sex

Table 3 details comparable analyses of the resilience scale and the single indicator of self-control as predictors of early sexual activity. This association was not linear. Instead, the pattern was J shaped with the most resilient boys and girls being more likely to have engaged in early sexual behaviour than the next most resilient group. This counter-intuitive finding (greatest resilience and early engagement in sex) was statistically significant only among girls and only after adjusting for SES and family support (RR= 0.43; 95% CI: 0.20–0.94). The least resilient adolescents, and particularly boys, were most likely to have had early sex. After adjusting for covariates, boys in the least resilient group were 3.57 times more likely (95% CI: 1.75–7.31) to have engaged in early sexual behaviour than were the most resilient. Lower self-control also aligned with increased early sex for boys and girls but the associations were not statistically significant after adjusting for family and community support and SES.

**Table 3:** Log-binomial regression estimating relative risk of engaging in early sexual activity (first had sex at ≤13 years old) by sex, grades 9 and 10

	*N* total	First had sex at age ≤13		Model 1^a^		Model 2^b^		Model 3^c^	
		*n*	(%)	RR	(95% CI)	RR	(95% CI)	RR	(95% CI)
Boys (*n*=3748)									
Resilience									
5 (highest)	697	26	(3.8)	1.00	–	1.00	–	1.00	–
4	957	22	(2.3)	0.60	(0.26–1.39)	0.61	(0.26–1.42)	0.55	(0.24–1.28)
3	1267	48	(3.8)	1.00	(0.55–1.81)	1.07	(0.58–1.98)	0.89	(0.46–1.74)
2	553	48	(8.7)	2.18	(1.01–4.70)	2.40	(1.05–5.52)	1.83	(0.82–4.08)
1 (lowest)	273	44	(16.2)	4.24	(2.10–8.56)	4.81	(2.29–10.10)	3.57	(1.75–7.31)
Self-control									
High	1681	65	(3.9)	1.00	–	1.00	–	1.00	–
Moderate	973	51	(5.3)	1.16	(0.68–1.86)	1.17	(0.68–1.99)	1.10	(0.64–1.90)
Low	1095	72	(6.6)	1.54	(1.04–2.30)	1.50	(0.99–2.29)	1.43	(0.91–2.26)
Girls (*n*=4090)								Model 4^d^	
Resilience									
5 (highest)	697	27	(3.9)	1.00	–	1.00	–	1.00	–
4	927	17	(1.9)	0.47	(0.20–1.10)	0.51	(0.23–1.15)	0.43	(0.20–0.94)
3	1423	36	(2.5)	0.63	(0.22–1.82)	0.73	(0.28–1.92)	0.53	(0.24–1.19)
2	735	54	(7.3)	1.81	(0.83–3.97)	2.19	(1.14–4.20)	1.35	(0.83–2.20)
1 (lowest)	307	27	(8.9)	2.23	(0.83–6.00)	2.76	(1.15–6.65)	1.54	(0.69–3.43)
Self-control									
High	1481	47	(3.2)	1.00	–	1.00	–	1.00	–
Moderate	1025	44	(4.3)	1.28	(0.97–1.98)	1.38	(1.04–1.81)	1.32	(0.99–1.77)
Low	1583	69	(4.4)	1.21	(0.57–2.58)	1.26	(0.58–2.74)	1.21	(0.56–2.62)

RR = relative risk; 95% CI = 95% confidence interval; adjusted for clustering by school and weighted.

a Model 1=unadjusted (resilience and self-control in the same model).

b Model 2=adjusted for socioeconomic status.

c Model 3=adjusted for socioeconomic status, family support, community support.

d Model 4=adjusted for socioeconomic status and family support.

## Discussion

The health impact of risk-taking on children and adolescents in North America, and the mortality burden of injury, persists despite preventive interventions and policies. We have examined individual (resilience) and environmental (family, school, social climate) circumstances in searching for modifiable personal assets that protect young people from engaging in risk-taking behaviours. As Prince-Embury found among 15–18 year olds, our 11–15 year old participants demonstrated that regardless of SES or family environment, there is a strong, dose-dependent relationship between traits that indicate resilience, and less risk-taking behaviour.^16^ Others have documented that such traits are amenable to intervention and augmentation, especially among children and youth.^17^ Together these findings suggest the possibility of a novel approach to prevention by identifying and strengthening individual assets that foster resilience.

To study the relationship between resilience and risk-taking behaviour we derived measures of both constructs from existing HBSC questions.^13–16^ There is general agreement that empathy, self-control, confidence/competence and optimism are key characteristics of resilience. There is no ‘gold standard’ for how to measure such attributes and existing resilience scales both overlap and diverge^29^ factor analysis enabled us to develop a theoretically sound proxy measure using five dataset questions. Self-control is often seen as central to resilience and, therefore, the question that most closely addressed this trait was also included as a separate measure.^30^ Our scaled measure of resilience, while rooted in past theory,^13–16^ was developed in an attempt to explore a novel topic using an existing database. As such, we did not have an optimal set of resilience indicators that might be incorporated into a scale in a confirmatory manner. We therefore used an exploratory factor analysis approach. Our factor loadings (all >0.30) and measure of internal consistency (Cronbach’s *α* = 0.63), while acceptable, were modest and suggest the need for further refinement of this scale with more focussed measures, moving forward.

Participants appeared resilient overall, yet significant variations emerged. Family, social and economic strengths aligned with greater resilience, while getting older had the opposite effect, particularly among girls. If adaptation to adversity aligns with resilience one might ask why those of lower SES had lower resilience. Others have demonstrated that while the adverse experiences of family and social disruption sometimes foster resilience, this may not be so for economic adversity.^31^ However, we have found that greater resilience scores align with healthier behaviour regardless of SES. Therefore, targeting those of lower SES, and particularly girls, with interventions to build resilience could be particularly advantageous.

The robust, incremental alignment between characteristics that suggest greater resilience and lower engagement in risk-taking behaviours is consistent with existing evidence, yet patterns identified in our analyses were, perhaps, more clear.^1^^,^^16^^,^^18^^,^^19^ The association between personal characteristics and risk-taking persisted even after controlling for external resources such as family, SES and supportive environments. This finding speaks to our fundamental question of whether greater individual assets can not only minimize risk-taking that leads to injury, morbidity and mortality but also override some of the negative impact of childhood adversity on behaviour. Our results suggest that building resilience by augmenting individual characteristics may be an effective approach to decreasing adolescent risk-taking. A few randomized controlled trials and a number of programme evaluations have demonstrated that such augmentation is possible.^11^^,^^17^^,^^32^ Further, our study shows that the path from lower social or economic adversity to risk-taking is not inevitable and is diverted in those adolescents with greater resilience.

Only the oldest participants (ages 13–15) were asked about early sexual activity. From the HBSC data it is impossible to determine whether reported sexual activity was consensual or forced. Although only a small proportion of the whole cohort had been sexually active prior to age 14, the least resilient were most likely to be among that group. The most resilient boys and girls reported early sexual activity in greater numbers than did the next group (boys) or two groups (girls). This pattern remained after adjusting for family and social circumstances. The J shaped, non-linear relationship between resilience and early sex may speak to the question of abusive rather than consensual activity. It is unclear whether early and forced sexual activity shatters resilience or if, instead, lower resilience underpins vulnerability. Conversely, consensual activity may arise from or lead to higher or lower self-confidence (one component of our resilience scale). This cannot be determined from HBSC data, alone. The alignment of self-control and early sexual activity is also difficult to interpret. Among boys there is a clear inverse relationship between the two that is no longer significant, after controlling for family and social supports. For girls, however, the association is not significant in any model. Despite the intuitive assumption that those with greater self-control will be more likely to defer sexual activity this is not indicated by the data. Perhaps this is because of uncertainties either around consensual sex or our measure of self-control.

Responses to the statement, ‘I have a hard time saying no’ were the measure of self-control. However, agreement with the statement could indicate a keenness to please rather than a lack of control. Ambiguity of associated outcomes may, therefore, speak as much to this indicator’s lack of sensitivity, as to the relationship between self-control and risk-taking.

As is true of any single observational study, despite a large and representative sample, we can only find associations between individual traits suggesting resilience and risk-taking. To determine whether this relationship is causal would require repeated similar observational findings or studies using experimental designs.

Although definitions of resilience consistently identify adversity as the precursor for adapting and thriving this does not, in any way, justify inequities or abuses that underlie adversity. Unfortunately, methods for tackling social and economic inequities and adversities at the individual or public health level are limited. However, our findings validate discussions between, e.g. primary care providers and patients about how bad experiences sometimes can become sources of strength.^33^ Clinicians and public health personnel might also lessen the harms of adversity by using or recommending available tools to build resilience among adolescents.^14^^,^^34^

Despite being the leading cause of adolescent death, minimizing injuries and other health hazards arising from risk-taking behaviours has been difficult. We have looked at whether there are malleable personal characteristics that diminish risk-taking. The traits of empathy, self-control, confidence/competence and optimism each foster resilience and have clear, incremental associations with adolescent risky behaviours regardless of social circumstances. Whether by asking the six questions used in this study or utilizing one of the validated resilience scales, health promoters could identify youth who would benefit from resilience building, and recommend or facilitate access to existing resources. The impact on risk-taking of such a preventive strategy may be greater than current population-level deterrents or physicians’ questions and statements about sex, drugs or violence.

## Funding

None of the authors received specific support for this research.

*Conflicts of Interest*: none declared.

**Key points**

- Despite population and individual level efforts, risky adolescent behaviours continue, bringing with them injury, unwanted pregnancy, addiction and mortality.
- We found that increments in individual, malleable traits like empathy, competence, optimism and self-confidence, all characteristics of resilience, are associated with diminished adolescent risk-taking.
- Least resilient youth were also most likely to engage in early sexual activity although this relationship was more complex.
- The resilience benefit occurs among girls and boys and regardless of SES, or measures of family and social adversity.
